# SNAP23 is essential for platelet and mast cell development and required in connective tissue mast cells for anaphylaxis

**DOI:** 10.1016/j.jbc.2021.100268

**Published:** 2021-01-08

**Authors:** Rodolfo A. Cardenas, Ricardo Gonzalez, Elizabeth Sanchez, Marco A. Ramos, Eduardo I. Cardenas, Alejandro I. Rodarte, Roberto J. Alcazar-Felix, Alejandro Isaza, Alan R. Burns, Ruth Heidelberger, Roberto Adachi

**Affiliations:** 1 Department of Pulmonary Medicine, The University of Texas MD Anderson Cancer Center, Houston, Texas, USA; 2 Tecnologico de Monterrey, Escuela de Medicina y Ciencias de la Salud, Monterrey, Nuevo León, Mexico; 3 Tecnologico de Monterrey, Escuela de Ingenieria y Ciencias, Monterrey, Nuevo León, Mexico; 4 College of Optometry, University of Houston, Houston, Texas, USA; 5 Department of Neurobiology and Anatomy, McGovern Medical School at The University of Texas Health Science Center at Houston, Houston, Texas, USA

**Keywords:** mast cell, platelets, exocytosis, SNAP23, SNARE proteins, allergy, anaphylaxis, AU, arbitrary units, B6, C57BL6/J mouse line, bm, basement membrane, BMMCs, bone-marrow-derived MCs, Δ*C*_m_, capacitance gain, CAE, chloroacetate esterase, *C*_m_, membrane capacitance, Cma1, chymase 1, CTMC, connective tissue MC, CTX, cholera toxin, DNP, 2,4-dinitrophenol, DT, diphtheria toxin, F, farad, FSC, forward scatter, Fura-2 AM, Fura-2-acetoxymethyl ester, *G*_m_, membrane conductance, *G*_S_, series conductance, GTPγS, guanosine 5′-3-*O*-(thio)triphosphate, HSA, human serum albumin, MC, mast cell, MFI, mean fluorescence intensity, MMC, mucosal MC, mMCP5/Mcpt5, mouse mast cell protease 5, MOV, mean object volume, Munc, mammalian homolog of *C. elegans* uncoordinated gene, OVA, ovalbumin, PCMCs, peritoneal-cell-derived MCs, Pf4, platelet factor 4, PMA, phorbol 12-myristate 13-acetate, RBL-2H3, rat basophilic leukemia cell line, S, siemens, SCF, stem cell factor, SNAP23, synaptosomal-associated protein of 23 KDa, SNARE, soluble N-ethylmaleimide-sensitive factor attachment protein receptors, Stx, syntaxin, Sv, surface density, Syt, synaptotagmin, VAMP, vesicle-associated membrane protein, Vv, volume density, Wsh, MC-deficient *Kit*^*W-sh/W-sh*^ mouse

## Abstract

Degranulation, a fundamental effector response from mast cells (MCs) and platelets, is an example of regulated exocytosis. This process is mediated by SNARE proteins and their regulators. We have previously shown that several of these proteins are essential for exocytosis in MCs and platelets. Here, we assessed the role of the SNARE protein SNAP23 using conditional knockout mice, in which SNAP23 was selectively deleted from either the megakaryocyte/platelet or connective tissue MC lineages. We found that removal of SNAP23 in platelets results in severe defects in degranulation of all three platelet secretory granule types, *i.e.*, alpha, dense, and lysosomal granules. The mutation also induces thrombocytopenia, abnormal platelet morphology and activation, and reduction in the number of alpha granules. Therefore, the degranulation defect might not be secondary to an intrinsic failure of the machinery mediating regulated exocytosis in platelets. When we removed SNAP23 expression in MCs, there was a complete developmental failure *in vitro* and *in vivo*. The developmental defects in platelets and MCs and the abnormal translocation of membrane proteins to the surface of platelets indicate that SNAP23 is also involved in constitutive exocytosis in these cells. The MC conditional deletant animals lacked connective tissue MCs, but their mucosal MCs were normal and expanded in response to an antigenic stimulus. We used this mouse to show that connective tissue MCs are required and mucosal MCs are not sufficient for an anaphylactic response.

Mast cells (MCs) modulate local and systemic inflammation through the release of inflammatory mediators (*e.g.*, histamine) stored in their large secretory granules (1). Platelets play key roles in hemostasis, thrombosis, and inflammation, which require release of alpha, dense, and lysosomal granules (2). Thus, degranulation is a main effector response from both cells. This process is an example of regulated exocytosis, in which cells release secretory vesicle contents through the fusion of vesicle and plasma membranes upon stimulation. This tightly regulated mechanism requires the interplay of SNARE (soluble N-ethylmaleimide-sensitive factor attachment protein receptor) proteins on the vesicle and plasma membranes and other SNARE-associated proteins (3).

One of the best studied models of regulated exocytosis is synaptic vesicle release, a process that depends on the formation of a quadruple-helix complex (SNARE complex) composed of the SNARE domains of vesicle-associated membrane protein (VAMP) 2 on the vesicle membrane and of syntaxin (Stx) 1 and synaptosomal-associated proteins of 25 KDa (SNAP25) on the plasma membrane. The last one donates two helices to the complex. Effective formation of SNARE complexes and fusion of these two membranes also require the participation of several other components, including synaptotagmin (Syt) 1, mammalian isoform of *C. elegans* uncoordinated gene 13 (Munc13) 1, and Munc18-1 (4).

Several homologs of these proteins participate in regulated exocytosis in other cell types, and we have studied the roles of Stx-3 and -4, Syt-2, Munc13-1 and -2, and Munc18-1, -2, and -3 in MCs and platelets. The MC provides a system to study single-vesicle and compound exocytosis at high resolution, and platelets allow the study of the differential regulation of three different exocytic compartments simultaneously (5, 6, 7, 8, 9, 10).

There are four mammalian homologs of SNAP25 (23, 25, 29 and 47) (11). In all of them, two SNARE domains are separated by a linker region. In SNAP23 and SNAP25, this linker contains a cysteine-rich sequence, and several of these cysteines are palmitoylated so the protein can partially penetrate membranes. It is postulated that SNAP29 and SNAP47, which lack palmitoylated cysteines, associate with membranes through their SNARE-partners (12, 13). SNAP23 is ubiquitously expressed and has been associated with exocytosis in many cell types (14, 15). SNAP25 has been mainly studied in neuronal and neuroendocrine exocytosis (16, 17). SNAP29 is ubiquitously expressed and localizes to intracellular membranes in the endosomal system and the Golgi (18). SNAP47 is found widespread on intracellular membranes, where it drives early secretory pathway export through the Golgi and autolysosome formation during autophagy (19, 20).

Our original goal was to study the roles of SNAP23 in platelet and MC exocytosis using conditional knockout animals. Deletion of this protein in the megakaryocyte/platelet line induced severe abnormalities in platelet number, morphology, and activation, which we quantify in this study. Although others have shown that deletion of SNAP23 results in an almost complete failure of platelet exocytosis, the platelet anomalies we describe here make it impossible to conclude that this is due to a pure exocytic defect. Furthermore, these abnormalities make any previously reported *in vivo* study uninterpretable (21).

We also show that in the absence of SNAP23, MCs fail to develop, and we took advantage of this. Based on ontogeny, distribution, expression patterns, histology, and responses to various stimuli, rodent MCs can be divided into connective tissue and mucosal MCs (22, 23), a subdivision that has an equivalent in humans (24, 25). Despite all these *in vitro* dissimilarities, we do not know how different the pathophysiological responses of these two subpopulations are *in vivo*. Our conditional deletion of SNAP23 eliminated connective tissue MCs, leaving mucosal MCs intact, and we use this model to show that the anaphylactic response depends almost exclusively on connective tissue MCs.

## Results

### Deletion of SNAP23 in MCs and platelets

**Figure 1:**
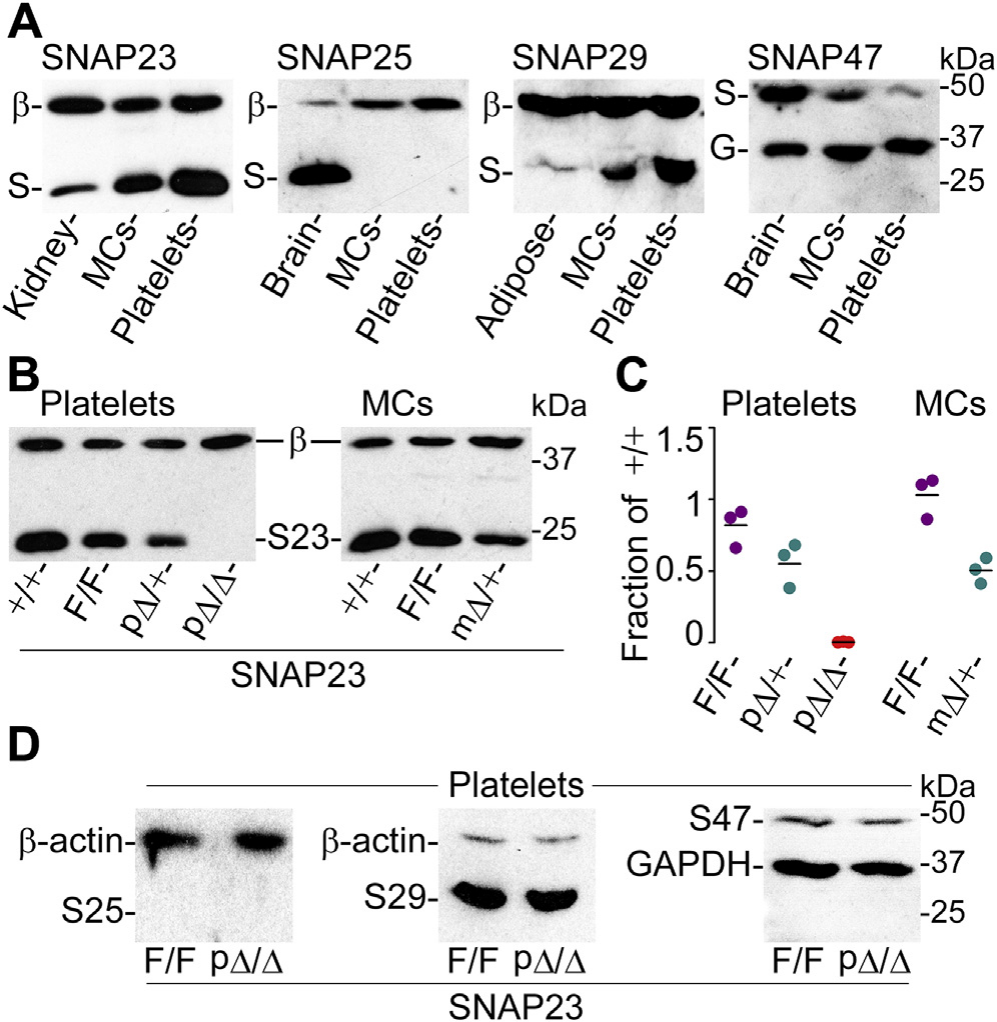
**Expression of SNAP23 and its homologs.** Shown are representative immunoblots. *A*, lysates of platelets and MCs from B6 mice probed with anti-SNAP23, 25, 29, and 47 antibodies. Other tissues were used as positive controls. *β*, β-actin; *G*, GAPDH; *kDa*, position of molecular weight standards; *S*, SNAP. *B*, lysates of platelets and MCs from different SNAP23 mutant mice probed with anti-SNAP23 antibody. *C*, densitometry ratios of SNAP23 relative to β-actin expressed as fractions of those ratios in SNAP23^+/+^ samples. *Dots*, individual values; *black line*, mean. *D*, lysates of platelets from SNAP23 mutant mice probed with anti-SNAP25, 29, and 47 antibodies.

In immunoblots of platelet and MCs lysates, we found that both cells express SNAP23, 29, and 47. We did not detect expression of SNAP25 despite loading ∼10x more total protein from MC and platelet lysates than from control tissues (Fig. 1*A*).

We crossed a conditional KO mouse, in which the *SNAP23* gene contains two loxP sequences (“floxed”; F allele; SNAP23^F/F^ mouse) (26), with two Cre-expressing mice. In one, the expression of Cre recombinase is driven in megakaryocytes and platelets by the promoter of platelet factor 4 (Pf4) (27), and SNAP23 is deleted in the platelets (pΔ allele) of their progeny (SNAP23^pΔ/Δ^ mouse). In the other, Cre is expressed in MCs under the control of the promoter of chymase 1 (Cma1), also known as mouse MC protease 5 (mMCP5/Mcpt5), deleting SNAP23 in a subset of MCs (mΔ allele) of the pups (SNAP23^mΔ/Δ^ mouse). Crossings of these conditional deletants with C57BL/6 (B6; SNAP23^+/+^) mice produced heterozygous SNAP23^pΔ/+^ and SNAP23^mΔ/+^ mice.

We demonstrated by immunoblots (Fig. 1, *B* and *C*) that we removed selectively SNAP23 in platelets (pΔ/Δ *versus* F/F), that insertion of the loxP sequences did not alter expression of SNAP23 (F/F *versus* +/+), and that SNAP23 expression is reduced by approximately half in heterozygotes (pΔ/+ and mΔ/+ *versus* F/F). Because deletion of SNAP23 in MCs altered the development of most MCs (see below), we could not recover enough MCs to run a lysate from mΔ/Δ mice). We also found that the removal of SNAP23 did not alter the expression of its homologs (Fig. 1*D*).

### Removal of SNAP23 alters platelet development, morphology, and activation

In our previous studies on platelet and MC exocytosis, we documented that removal of proteins crucial for this process did not affect cell ultrastructure, development or activation, a fundamental step before studying any functional defect (5, 6, 7, 8, 9, 10, 28).

**Figure 2:**
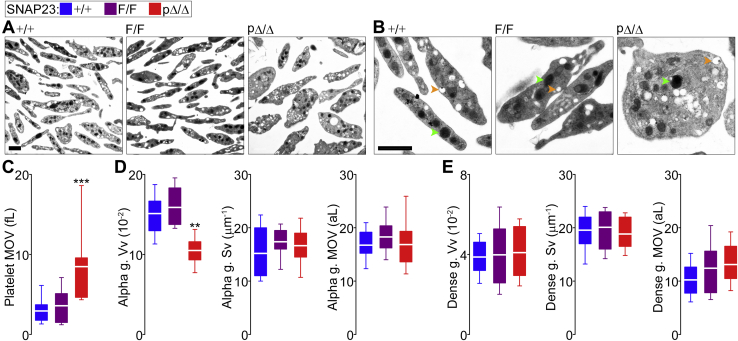
**Abnormal platelet morphology in the absence of SNAP23.** Color legend on top applies to all graphs. *A* and *B*, representative EM images at two magnifications of platelets from different SNAP23 mutant mice. *Arrowheads* point examples of granules: *green*, alpha granule; *orange*, dense granule. *Scale bars* = 1 μm. *C*, mean object volume (MOV) of platelet EM profiles. *n* = 21. *D* and *E*, volume density (Vv), surface density (Sv) and MOV of alpha granules and dense granules, respectively. *n* = 11 to 21. *White line*, mean; *box*, 25th to 75th percentile; *whiskers*, 5th to 95th percentile. ∗∗*p* ≤ 0.01, ∗∗∗*p* ≤ 0.001; all compared to F/F.

When we studied the platelets from our mutant mice under EM, we observed that SNAP23-deficient platelets appeared to be larger and to have fewer alpha granules (Fig. 2, *A* and *B*). To quantify these qualitative changes, we applied stereology. We obtained the mean object volume (MOV; average volume of a sample of objects independent of their shape) (29) of platelets and their granules. Platelets from SNAP23^pΔ/Δ^ mice had almost triple the MOV of platelets from SNAP23^F/F^ mice (Fig. 2*C*). The volume density of alpha granules (Vv; volume of platelets occupied by alpha granules) was significantly decreased in platelets lacking SNAP23, without alterations in surface density (Sv; relationship of the surface of a granule to its volume) and MOV (Fig. 2*D*). This combination of stereological parameters is only possible when there is a significant decrease in the number of the quantified object (30), in this case alpha granules per platelet. We calculated the same parameters for dense granules and saw that none was affected by the absence of SNAP23 (Fig. 2*E*).

**Figure 3:**
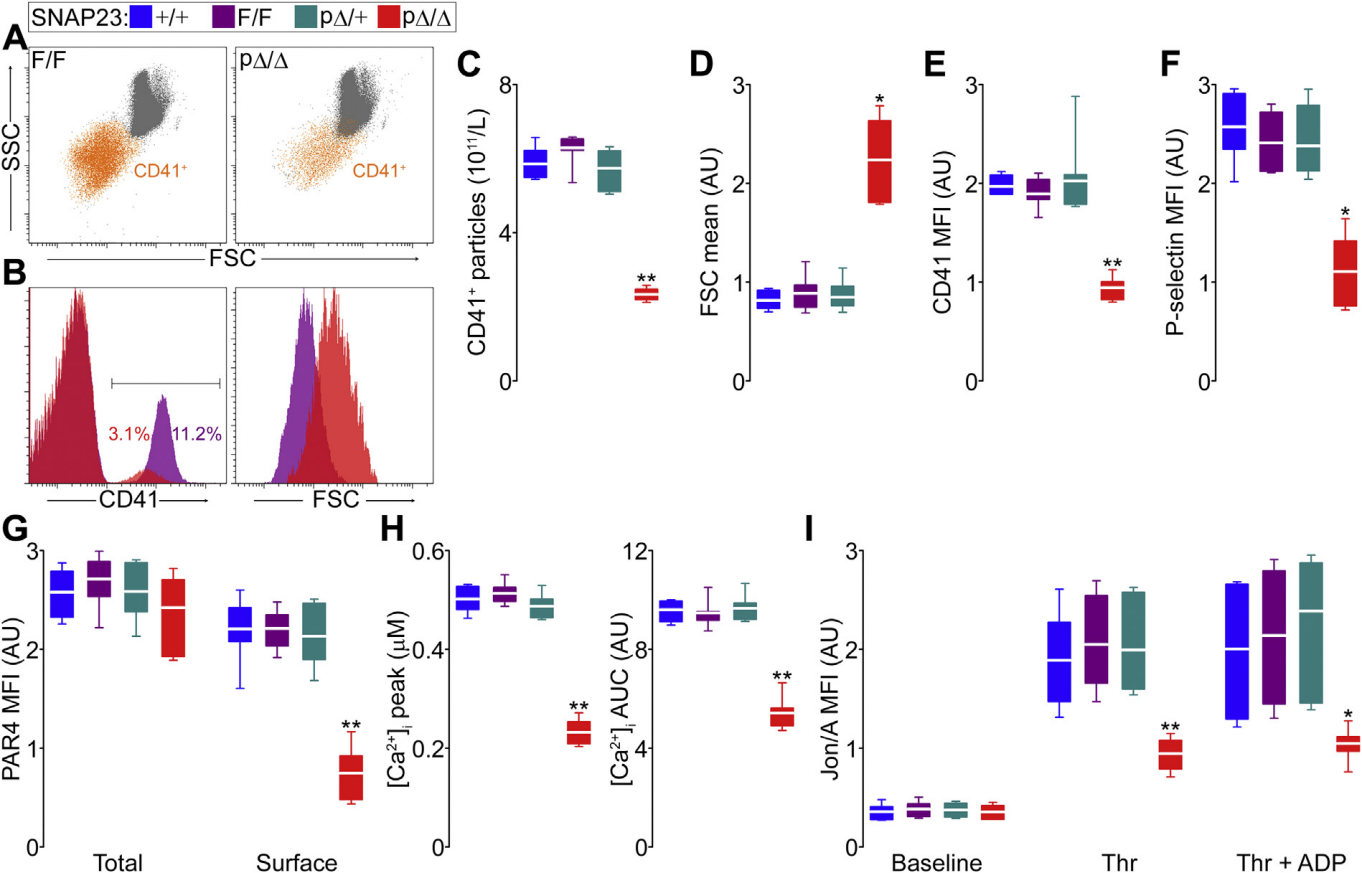
**Abnormal number, expression of surface proteins, and activation of platelets in the absence of SNAP23.** Color legend on top applies to all graphs. Whole-blood samples were loaded with a predetermined number of fluorescent beads, labeled with fluorescent antibodies against CD41 and P-selectin, and studied under flow cytometry. *A*, representative scattergrams of samples from SNAP23^F/F^ and SNAP23^pΔ/Δ^ littermates. *FSC*, forward scatter; *SSC*, side scatter; *orange dots*, CD41^+^ particles. *B*, representative histograms comparing CD41 fluorescence and FSC in the same F/F and pΔ/Δ samples. *C*, concentration of platelets (CD41^+^ particles) in whole-blood samples using fluorescent beads to calculate the original volume. *n* = 7; applies to *C*–*F*. *D*, difference in particle sizes estimated using the mean FSC values of CD41^+^ particles. *E*, mean fluorescence intensity (MFI) for CD41 in CD41^+^ particles. *F*, MFI for P-selectin in CD41^+^ particles. *G*, MFI for PAR4 in fixed and permeabilized platelets (total) or intact platelets (surface). *n* = 11. *H*, peak and area under the curve (AUC) over time of intracellular calcium concentrations ([Ca^2+^]_i_) measured by ratiometry in Fura-2 AM-loaded platelets activated with thrombin (0.1 U/ml). *n* = 12. *I*, MFI of intact platelets labeled with an antibody (Jon/A) against the activated conformation of α_IIb_β_3_ before and after exposure to thrombin (Thr; 0.1 U/ml) with and without ADP (10 μM). *n* = 7. *White line*, mean; *box*, 25th to 75th percentile; *whiskers*, 5th to 95th percentile. ∗*p* ≤ 0.05, ∗∗*p* ≤ 0.01; all compared to F/F.

By analyzing blood samples in an automated counter, we found that SNAP23^pΔ/Δ^ mice were thrombocytopenic, having only ∼40% of the circulating platelets found in their littermate controls (Fig. S1). Red blood cell, white blood cell, and differential counts were not affected. Because the larger platelets in these mutant mice could cause spurious results in an automated counter, we confirmed our findings by flow cytometry, identifying platelets as CD41^+^ particles. In scattergrams from pΔ/Δ samples, the population of CD41^+^ particles was less dense and migrated toward a higher forward scatter (FSC) (Fig. 3*A*). Histograms showed a reduced fraction of CD41^+^ particles, which had a larger FSC (Fig. 3*B*). We used fluorescent beads to calculate the original volume of each sample and confirmed that SNAP23^pΔ/Δ^ mice were thrombocytopenic (Fig. 3*C*). FSC correlates with particle size, and it was significantly increased in the CD41^+^ fraction of pΔ/Δ samples, confirming that platelets lacking SNAP23 were larger (Fig. 3*D*).

We quantified the lower intensity of CD41 on platelets from SNAP23^pΔ/Δ^ mice (Fig. 3*B*) and found that intact platelets from these mutant mice express only ∼1/2 of this integrin on their surface (Fig. 3*E*). Other platelet surface markers, such as P-selectin, were also reduced in pΔ/Δ platelets (Fig. 3*F*). Because we were planning to use thrombin as an agonist in our experiments, we measured expression of PAR4, the main receptor for thrombin in mouse platelets (31). We found that, although total levels of PAR4 were similar in permeabilized platelets from all genotypes, there was a significant decrease on the expression of PAR4 on the surface of intact platelets lacking SNAP23 (Fig. 3*G*), pointing to a trafficking defect.

We then measured the rise of the intracellular concentration of Ca^2+^ ([Ca^2+^]_i_) by ratiometry in platelets exposed to thrombin. We found that both the peak [Ca^2+^]_i_ and the area under the curve (AUC) of [Ca^2+^]_i_ over time were severely decreased in SNAP23-deficient platelets (Fig. 3*H*).

Upon stimulation, additional integrin α_IIb_β_3_ is translocated from intracellular compartments to the plasma membrane. Also, this integrin undergoes a conformational change from a low affinity to a high-affinity state. This transformation, which can be detected by specific antibodies (Jon/A), is used as a marker of platelet activation (32, 33). When we compared levels of activated α_IIb_β_3_ on platelets exposed to thrombin, we detected a significant deficiency in the absence of SNAP23. This defect could not be rescued by adding ADP as a second agonist (Fig. 3*I*).

These results show that deletion of SNAP23 in platelets drastically affects platelet numbers, levels of plasma membrane proteins, activation, morphology, and generation of alpha granules.

### Severe exocytic defects in platelets in the absence of SNAP23

We decided to test the effects of these alterations on platelet exocytosis. We assessed markers from the three types of platelet granules: increase in extracellular ATP for dense granules, and translocation of P-selectin and LAMP-1 from intracellular compartments to the plasma membrane for alpha granules and lysosomal granules, respectively (34, 35, 36).

**Figure 4:**
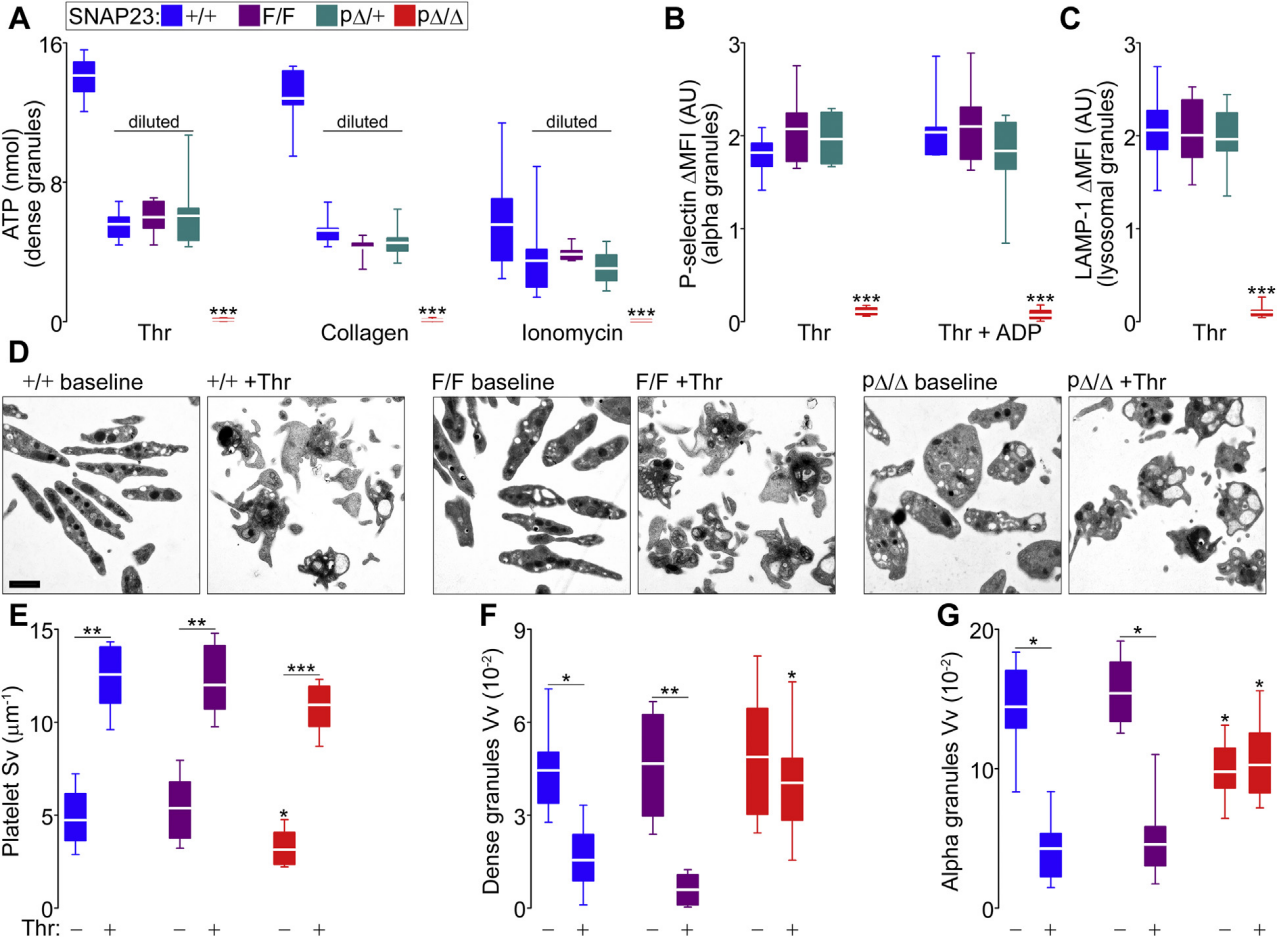
**Abnormal platelet exocytosis in the absence of SNAP23.** Color legend on top applies to all graphs. *A*, whole-blood samples were exposed to thrombin (Thr; 0.1 U/ml), collagen (10 μg/ml), or ionomycin (10 μM), and dense granule secretion was assessed by increase in extracellular ATP (luminometry). +/+, F/F and pΔ/+ samples were diluted (∼1:2.5) to match the platelet count in pΔ/Δ samples; an undiluted +/+ sample was used as a control. *n* = 7. *B* and *C*, washed platelets labeled with fluorescent antibodies against P-selectin and LAMP-1 studied under flow cytometry before and after stimulation with thrombin with or without ADP (10 μM), while recording the difference in mean fluorescence intensity (ΔMFI). Alpha and lysosomal granule secretion were assessed by increase in platelet surface translocation of P-selectin (*B*; *n* = 7) or LAMP-1 (*C*; *n* = 10), respectively. *D*, representative EM profiles of platelets before and after activation with thrombin. *Scale bar* = 1 μm. *E*, surface density (Sv) of platelet EM profiles before and after activation. *n* = 12; applies to *E*–*G*. *F* and *G*, volume density (Vv) of dense and alpha granules, respectively, before and after activation. *White line*, mean; *box*, 25th to 75th percentile; *whiskers*, 5th to 95th percentile. ∗*p* ≤ 0.05, ∗∗*p* ≤ 0.01, ∗∗∗*p* ≤ 0.001; all compared to F/F unless otherwise specified (horizontal lines).

For ATP measurements, we diluted whole-blood samples from SNAP23^+/+^, SNAP23^F/F^, and SNAP23^pΔ/+^ mice ∼1:2.5 to match the low number of circulating platelets found in SNAP23^pΔ/Δ^ mice. Upon exposure to thrombin, we observed a profound exocytic defect in dense granule exocytosis, which was reproduced when using collagen as a different agonist (Fig. 4*A*). We used ionomycin to bypass the defective increase in [Ca^2+^]_i_ we observed in SNAP23-deficient platelets stimulated with thrombin. A concentration of ionomycin that was sufficient to induce ATP release in samples from +/+, F/F and pΔ/+ mice did not induce secretion in those from pΔ/Δ mice.

We also detected an almost complete failure in alpha and lysosomal granule release (Fig. 4, *B* and *C*). Because ADP released from dense granules acts in a paracrine fashion to activate platelets (9), we wondered if some of these results were a downstream effect of deficient dense granule release, but addition of exogenous ADP to our experiments did not make a difference (Fig. 4*B*).

These secretion assays cannot assess intracellular events, so we compared resting and thrombin-activated platelets under EM and quantified changes by stereology. Qualitatively, platelets from all genotypes seemed to undergo morphologic changes: adopting irregular shapes with protrusion of pseudopodia (37). However, pΔ/Δ platelets seem to retain their granules despite stimulation (Fig. 4*D*). Geometry of an object affects its Sv: the smaller, less spherical, and more irregular an object, the larger its Sv (29). The smaller Sv at baseline in platelets lacking SNAP23 reflects that these are rounder and larger, as demonstrated above. Despite that, platelets of all genotypes increased their Sv upon stimulation, quantifying their marked shape deformation (Fig. 4*E*). The prominent loss of intracellular dense granules in activated SNAP23-sufficient platelets was quantified as a significant fall in their Vv, a change that was absent in pΔ/Δ platelets (Fig. 4*F*). The same was observed in alpha granules, the smaller number of granules present at baseline in SNAP23-deficient platelets were not released after stimulation (Fig. 4*G*).

Due to the thrombocytopenia, altered expression of plasma membrane receptors, significant changes in platelet structure, and faulty activation present when SNAP23 was removed, it made no sense to proceed to *in vivo* studies of thrombosis and hemostasis as we have done before (9, 10), since they would not lead to any clear conclusion.

### SNAP23 is required for MC development

**Figure 5:**
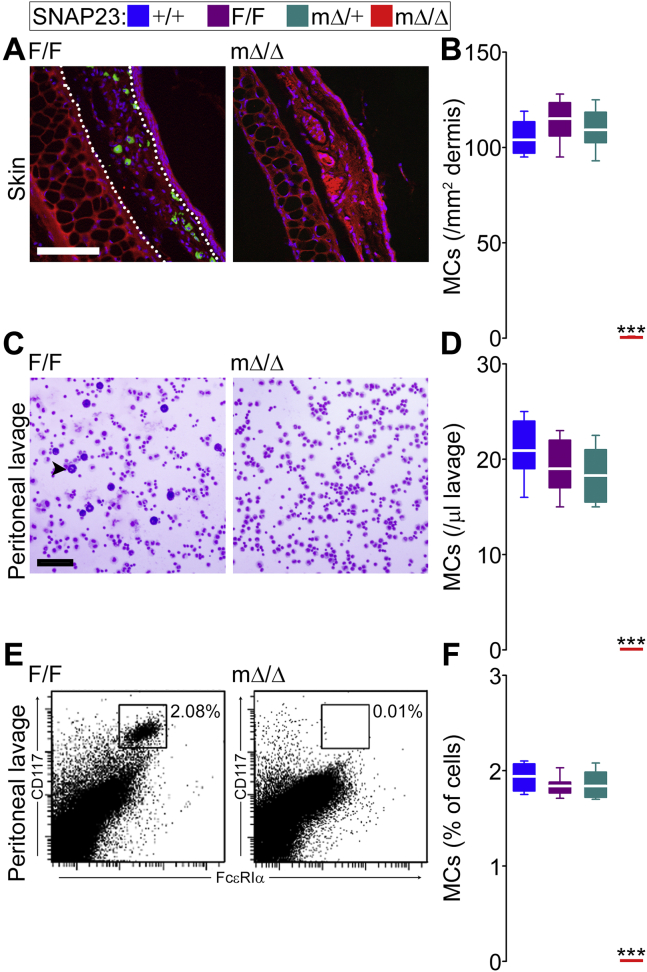
**Absence of connective tissue MCs after selective deletion of SNAP23.** Color legend on top applies to all graphs. *A*, representative images of ear sections stained with FITC-avidin (*green*) and Hoechst 3342 (*blue*); autofluorescence in the *red channel* delimited the dermis as the area between the epidermis and the auricular cartilage (*dotted line*). *Scale bar* = 100 μm. *B*, quantification of MCs (Hoechst^+^ nucleus surrounded by FITC^+^ granules) per area or dermis. *n* = 9. *C*, representative cytospins of peritoneal lavages stained with Wright-Giemsa. *Arrowhead*, example of a metachromatic MC. *Scale bar* = 100 μm. *D*, number of MCs (large cells with metachromatic granules) per volume of lavage. *n* = 10. *E*, representative scatter plot of flow cytometry of peritoneal lavage cells labeled with fluorescent antibodies against Kit/CD117 and FcεRIα. *Inset square*, double-positive cells. *F*, average fraction of MCs (double-positive cells) in the lavages. *n* = 10. *White line*, mean; *box*, 25th to 75th percentile; *whiskers*, 5th to 95th percentile. ∗∗∗*p* ≤ 0.001; all compared to F/F.

We wanted to test if similar morphological and functional defects could be produced by the deletion of SNAP23 in MCs. Connective tissue MCs are abundant in the dermis and peritoneal cavity, and we observed many MCs in SNAP23-sufficient animals. Avidin has high affinity for anionic compounds, and the most negatively charged molecule in mammals is heparin (38), so we use it to label MC granules in tissues (39). We failed to identify any MC in the dermis of SNAP23^mΔ/Δ^ mice (Fig. 5, *A* and *B*). In the same animals, we could not visualize any metachromatic cell in peritoneal lavages (Fig. 5, *C* and *D*). Because both of those techniques rely on staining of MC granules, we could be missing immature MCs or MCs with defective granule biogenesis, so we also used MC surface markers. MC survival and function depend on their expression of Kit/CD117 and FcεRI, and the simultaneous detection of both identifies a MC (40). By flow cytometry, the number of double-positive cells we could identify in peritoneal lavages from SNAP23^mΔ/Δ^ mice was almost zero (Fig. 5, *E* and *F*).

**Figure 6:**
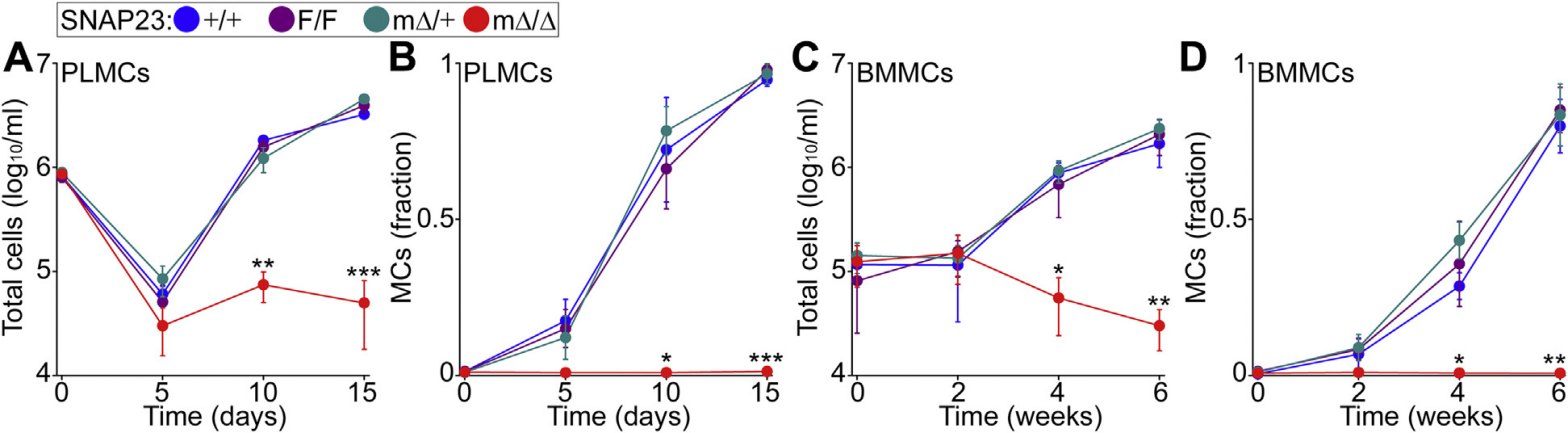
**Failure to differentiate MCs *in vitro* in the absence of SNAP23.** Color legend on top applies to all graphs. Peritoneal lavage cells (*A* and *B*) and bone marrow cells (*C* and *D*) were cultured in media enriched with IL-3 and SCF. Total number of cells (*A* and *C*) and fraction of MCs (CD117^+^ FcεRIα^+^ double-positive cells by flow cytometry) (*B* and *D*) in cultures over time. *n* = 5. *Dots*, mean; *error bar*, S.E. ∗*p* ≤ 0.05, ∗∗*p* ≤ 0.01, ∗∗∗*p* ≤ 0.001; all compared to F/F.

The lack of MCs in several tissues from SNAP23^mΔ/Δ^ mice could be due to a decrease in MC survival or production. To address the latter we tried to force MC differentiation *in vitro* using two primary cultures systems: peritoneal-cell-derived MCs (PCMCs) (41) and bone-marrow-derived MCs (BMMCs) (42, 43). Although both systems depend on IL-3 and stem cell factor (SCF), in our hands, PCMC cultures produce more MCs per animal, at a higher fraction, and in a shorter amount of time. We observed the typical time course in samples obtained from control and heterozygote mice, with an initial dip in total cell number followed by enrichment of MCs, but samples from SNAP23^mΔ/Δ^ animals had poor survival, and we could barely isolate CD177 and FcεRIα double-positive cells from them (Fig. 6). The few cells remaining in those cultures appeared to be fibroblasts.

These results indicate that SNAP23 is required for MC development and that it is haplosufficient for this function.

### Partial expression of SNAP23 is enough to sustain MC exocytosis

In other cells, partial deficiency of SNAP23 expression affects exocytosis (44) but in all our assays we could not document evidence of haploinsufficiency in SNAP23^pΔ/+^ or SNAP23^mΔ/+^ mice. In our studies on Munc13-4 deficiency, only the use of electrophysiology at a high resolution allowed us to see a defect proportional to the level of protein expression (6). Measurements of plasma membrane capacitance (*C*_m_) in single-peritoneal MCs using the whole-cell patch clamp configuration can identify single-fusion events (45). So, we decided to apply this method to study freshly harvested peritoneal MCs from SNAP23^mΔ/+^ mice. Because of the developmental defect, we were not able to study MCs from SNAP23^mΔ/Δ^ mice.

**Figure 7:**
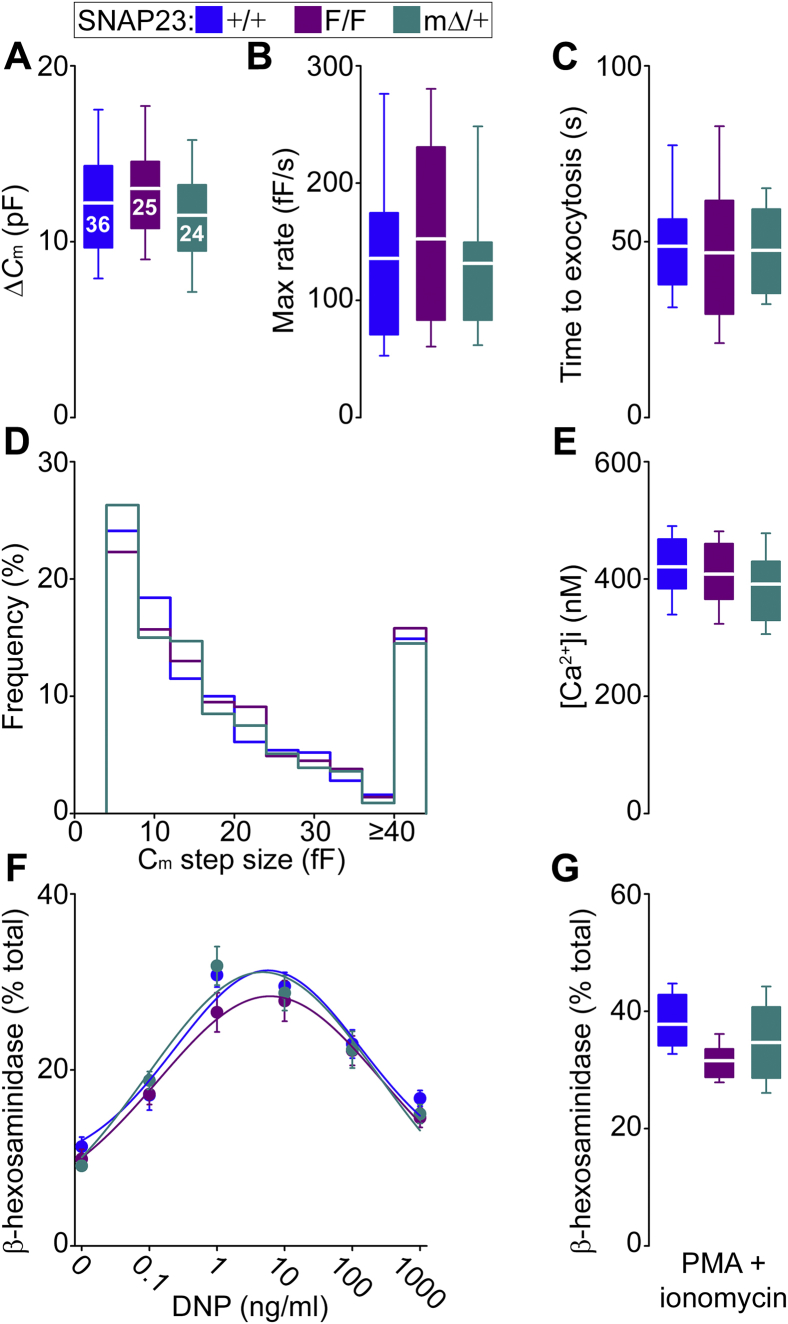
**SNAP23 is haplosufficient to sustain MC exocytosis.** Color legend on top applies to all graphs. *A*–*E*, freshly isolated single peritoneal MCs were studied by whole-cell patch clamp while exocytosis was induced by intracellular dialysis of Ca^2+^ and GTPγS. *A*, total gain in plasma membrane capacitance (Δ*C*_m_). *Number inside boxes* in *A*, number of cells studied obtained from seven animals of each genotype; applies to *A*–*E*. *B*, rate of exocytosis between 40% and 60% of total Δ*C*_m_. *C*, time interval between cell access and beginning of the exocytic burst. *D*, frequency distribution of *C*_m_ step sizes. *E*, steady-state [Ca^2+^]_i_ monitored by Fura-2 ratiometry. *F* and *G*, PCMCs were sensitized with anti-DNP IgE and then exposed to different concentrations of DNP-HSA (*F*) or to PMA and ionomycin (*G*). *n* = 7. Degranulation was measured as the released fraction of total cell β-hexosaminidase. *Dots* and *white line*, mean; *error bars*, S.E.; *box*, 25th to 75th percentile; *whiskers*, 5th to 95th percentile. No significant differences were detected.

The *C*_m_ of a cell is proportional to its surface area, and it is expected to increase (Δ*C*_m_) upon addition of vesicle membrane to the plasma membrane during exocytosis (46). Intracellular dialysis of GTPγS and Ca^2+^ through the patch pipette induces almost complete MC degranulation (47). The baseline *C*_m_ (not shown) and Δ*C*_m_ (Fig. 7*A*) were not different among +/+, F/F, and mΔ/+ MCs.

Activated MCs undergo single-vesicle and compound exocytosis (48), and we have shown that the rate of exocytosis is very sensitive to alterations in the latter (8). We have also demonstrated that prolongation of the time between stimulus (cell penetration) and response (exocytic burst) is a marker of defective exocytosis (6). We did not detect any defect in heterozygote MCs in both parameters (Fig. 7, *B* and *C*). We also measured the amplitude of individual *C*_m_ steps, which are proportional to the size of the secretory vesicles fusing with the plasma membrane (45), and found no differences (Fig. 7*D*). We controlled for the stimulus used and achieved the same [Ca^2+^]_i_ in all cells (Fig. 7*E*).

Finally, we assessed degranulation in populations of MCs using two stimuli, FcεRI cross-linking and PMA with ionomycin. We observed no differences in the bell-shaped dose–response curve to the first or in the stronger response to the second (Fig. 7, *F* and *G*).

### Mucosal MCs survive in our model

**Figure 8:**
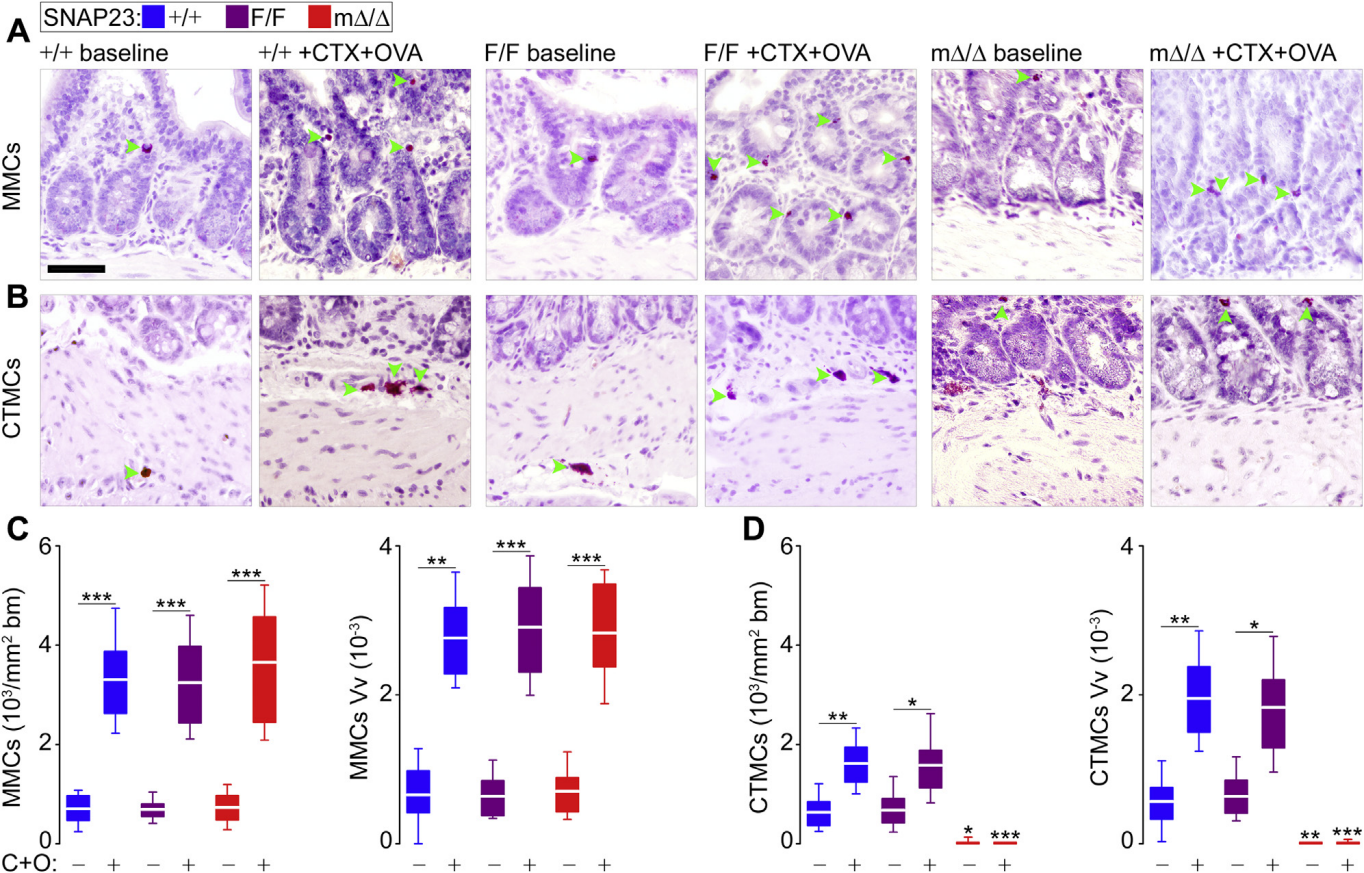
**Mucosal MCs survive in SNAP23**^**mΔ/Δ**^**mice and expand *in vivo*.** Color legend on top applies to all graphs. Samples of intestine were collected from mice before and after administration of cholera toxin (CTX) and ovalbumin (OVA) by gavage (C + O). *A* and *B*, representative histochemical images of mucosal MCs (MMCs) and connective tissue MCs (CTMCs), respectively. Note that in samples from mΔ/Δ mice, there are no visible CTMCs while MMCs are still present. *Green arrowheads*, red precipitate indicates CAE activity and labels MCs. *Scale bar* = 50 μm. *C* and *D*, quantification of MCs using the number of MCs per area of basement membrane (bm) and the volume fraction (Vv) of MCs in the host tissue. MCs were identified as mucosal (*C*) or connective tissue (*D*) MCs using the bm as the boundary. *n* = 33 to 40 images from 7 animals. *White line*, mean; *box*, 25th to 75th percentile; *whiskers*, 5th to 95th percentile. ∗*p* ≤ 0.05, ∗∗*p* ≤ 0.01, ∗∗∗*p* ≤ 0.001; all compared to F/F unless otherwise specified (horizontal lines).

Because of the expression pattern of Cma1/mMCP5, Cre is not expressed in mucosal MCs in Cma1-cre mice (49, 50), so we postulated that mucosal MCs should be intact in SNAP23^mΔ/Δ^ mice. Using chloroacetate esterase (CAE) to identify MCs in intestinal tissue samples, we observed MCs interspaced within the intestinal epithelium in mice of all genotypes (Fig. 8*A*), while there was an absence of MCs in the submucosa and other intestinal connective tissues from SNAP23^mΔ/Δ^ mice (Fig. 8*B*).

Furthermore, we could force these mucosal MCs to expand *in vivo* using a model of allergic enteritis. In this model, administration of cholera toxin (CTX) and ovalbumin (OVA) by gavage induces proliferation of mucosal and connective tissue MCs (51). Exposure to CTX alone did not alter the number of MCs (Fig. S2). Using the basement membrane as boundary, we quantified the number of mucosal and connective tissue MCs by two methods: by normalizing the results per area of basement membrane, and by calculating the Vv of MCs per volume of host tissue (Fig. 8, *C* and *D*). In +/+ and F/F mice we found the expected expansion of mucosal and connective tissue MCs, which was more marked in the former. In SNAP23^mΔ/Δ^ mice, mucosal MCs expanded to comparable levels but the connective tissue MCs remained almost completely absent. The few (a total of five in all the sections) MCs classified as connective tissue MCs in SNAP23^mΔ/Δ^ mice had partial overlap with the basement membrane, and by protocol they had to be counted as connective tissue MCs.

These results confirm the absence of connective tissue MCs in SNAP23^mΔ/Δ^ mice, even with antigenic stimulation. On the other hand, mucosal MCs numbers and proliferation are preserved in these mutants.

### Mucosal MCs are not sufficient to elicit anaphylaxis

Given that mucosal MCs were preserved despite the lack of connective tissue MCs in SNAP23^mΔ/Δ^ mice, we wanted to test if this subpopulation of MCs was able to mediate an anaphylactic reaction. We used a model of passive systemic anaphylaxis in which animals are sensitized with anti-DNP IgE, challenged with DNP, and develop hypothermia as a manifestation of anaphylaxis (52).

**Figure 9:**
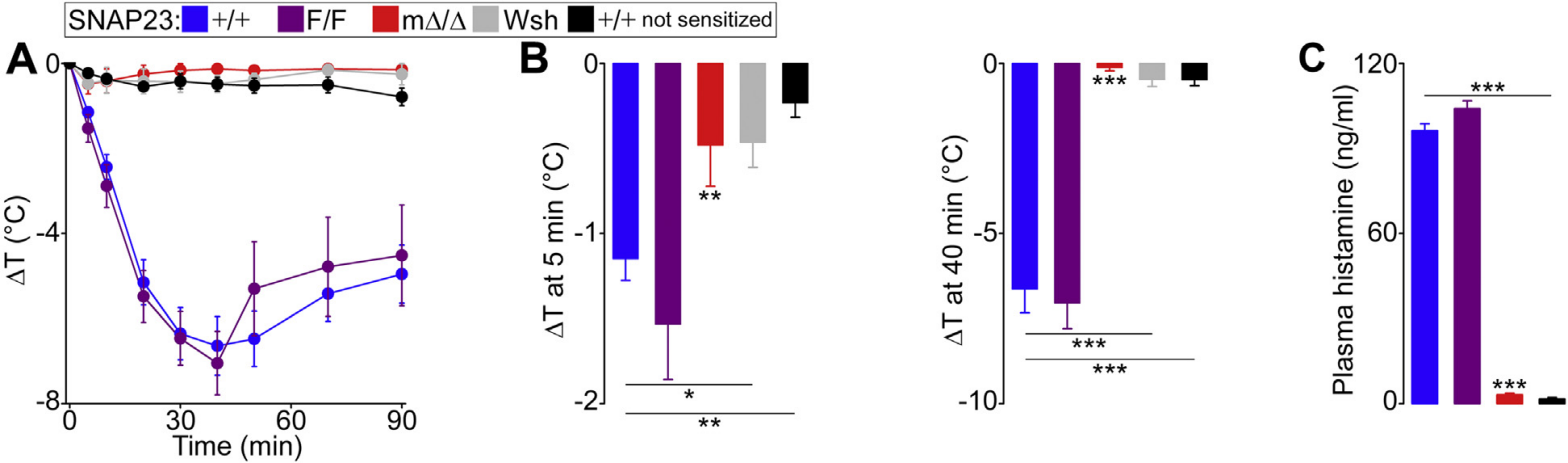
**Lack of anaphylactic response in the absence of connective tissue MCs.** Color legend on top applies to all graphs. MC-deficient *Kit*^*W-sh/W-sh*^ (Wsh) and SNAP23 mutant mice were sensitized i.p. with anti-DNP IgE and challenged i.p. with DNP-HSA, except a group of +/+ mice that were challenged but not sensitized. *A*, changes in body core temperature (ΔT). *B*, ΔT at 5 and 40 min postchallenge. *C*, plasma histamine measured by ELISA 15 min after challenge. *n* = 6. *Dots* and *bars*, mean; *error bars*, S.E. ∗*p* ≤ 0.05, ∗∗*p* ≤ 0.01, ∗∗∗*p* ≤ 0.001; all compared to F/F unless otherwise specified (horizontal lines).

We introduced unsensitized but challenged +/+ mice as controls to show that challenge alone does not induce hypothermia (Fig. 9*A*). As a second control, we used sensitized and challenged MC-deficient *Kit*^*W-sh/W-sh*^ (Wsh) mice and confirmed that this reaction is MC-dependent. While we observed the expected hypothermic response in SNAP23-sufficient mice, SNAP23^mΔ/Δ^ mice had negligible changes in their body core temperature (ΔT) at early and late time points (Fig. 9*B*). Actually, their response was indistinguishable from that of unsensitized or MC-deficient animals, and this correlated with a lack of circulating histamine after challenge (Fig. 9*C*).

These results indicate that connective tissue MCs are the main drivers of this anaphylactic response.

## Discussion

Although selective deletion of SNAP23 had a marked impact on development of platelets and MCs, it was more severe in the latter. The possibility that the surviving platelets underwent incomplete recombination, sparing one of the *SNAP23* alleles, is not supported by our findings that SNAP23 was undetectable in SNAP23^pΔ/Δ^ platelets (Fig. 1) and that SNAP23^pΔ/+^ mice had normal number of platelets, expression of surface receptors, and markers of activation (Fig. 3). Platelets and MCs also express the same SNAP23 homologs (SNAP29 and 47), and there is no alteration in their expression in the absence of SNAP23 (Fig. 1). Thus, while SNAP23 seems to be absolutely required for MC development, the dependency is only partial for platelets.

The surviving platelets in SNAP23^pΔ/Δ^ mice were very abnormal. They had almost triple the volume and were rounder, as indicated by the higher MOV and lower Sv compared with those from SNAP23^F/F^ littermates (Figs. 2 and 4). There was also a marked reduction in the number of alpha granules in the absence of SNAP23, but this was not a generalized failure in the production of secretory vesicles, because the parameters for dense granules were intact (Fig. 2).

Although secretory granules are released *via* regulated exocytosis, the transport of surface proteins in resting cells depends on constitutive exocytosis (53, 54). The reduction of several plasma membrane proteins in SNAP23-deficient platelets, particularly that of PAR4 in the presence of normal total cell levels (Fig. 3), suggests that this SNARE protein is involved in both exocytic mechanisms. This impaired export of receptors to the plasma membrane induced defective platelet activation (Fig. 3).

We have reported that deletion of Munc13-4 and Munc18-2 affects specifically platelet degranulation, based on the fact that the deletions did not perturb platelet development and activation (9, 10). Although it has been suggested that the removal of SNAP23 renders the exocytic machinery of platelets nonfunctional (21), the severe alterations in platelet morphology and activation we observed in SNAP23-deficient platelets prevent us from drawing any clean conclusion from exocytic assays. Add to that significant thrombocytopenia, and *in vivo* assays reported by others (21) become uninterpretable.

Despite all that, the severity of the exocytic defects we detected seems disproportionate to the structural and activation changes. Although the rise in [Ca^2+^]_i_ was reduced (Fig. 3), it was still sufficient to provoke shape changes in the absence of SNAP23 (Fig. 4). Despite the fact that there was a reduction in the number but not the absence of alpha granules (Fig. 2), we could not detect translocation of P-selectin upon activation (Fig. 4). Finally, the baseline number of dense granules was normal (Fig. 2), but secretion of those granules was undetectable by stereology and luminometry, even when using a Ca^2+^ ionophore to bypass defective thrombin signaling (Fig. 4). Therefore, platelet secretion depends on SNAP23, but it is not possible to quantify how much of the secretory defect in platelets lacking SNAP23 is due to an intrinsic exocytic defect or to abnormalities in platelet development and signaling. This will require an approach other than genetic deletion.

Cma1/mMCP5/Mcpt5 was identified as a protease expressed in connective tissue but not mucosal MCs (55), and expression of Cre recombinase in Cma1-cre mice follows this pattern (49, 50). So, we were not surprised that connective tissue MCs were affected more severely than mucosal MCs in our studies. (Figs. 5 and 8). Cma1/mMCP5/Mcpt5 is also expressed in *in vitro* differentiated MCs (56). Our failure to obtain PCMCs and BMMCs from SNAP23^mΔ/Δ^ mice (Fig. 6) indicates that SNAP23 is required at an early and fundamental step in MC development.

The absence of connective tissue MCs and the inability to differentiate MCs *in vitro* in mice lacking SNAP23 were very different from what we found when we removed expression of other proteins involved in MC exocytosis such as Syt-2, Munc13-4, Munc18-2, and Stx-3, none of which resulted in abnormalities in MC development or structure (5, 6, 7, 8). Given that constitutive exocytosis is also required for cell growth and development (53), this is additional evidence that SNAP23 might be involved in this process (57).

Previous evidence of the roles of SNAP23 in MC exocytosis comes from work in RBL-2H3 cells. By using expression of mutant SNAP23 constructs, they show that the participation of SNAP23 in exocytosis requires its association to the plasma membrane through its cystein-rich domain and dynamic phosphorylation at specific residues (58, 59, 60).

The lack of connective tissue MCs, PCMCs, and BMMCs deprived us of the most reliable sources of MCs for our exocytosis assays. Previous attempts in our lab failed to obtain mucosal MCs from gastric and intestinal epithelium that were intact enough to form the patch pipette gigaseal required to obtain *C*_m_ readings. Thus, we were unable to study MC exocytosis in SNAP23^mΔ/Δ^ mice. When we deleted expression of Munc13-4 in MCs, we observed a dose response between expression of this protein in MCs and the efficiency of regulated exocytosis, but only when using high-resolution assays such as single-cell electrophysiology and stereology of EM images (6). Nevertheless, when we applied those methods to study MCs form SNAP23^mΔ/+^ mice that have ∼50% expression of this protein (Fig. 1), we found no phenotype (Fig. 7). Therefore, like in the case of Munc18-2 and Stx-3 (7, 8), partial expression of SNAP23 is enough to sustain a full exocytic response in MCs. This differs from airway epithelial cells, where heterozygous SNAP23 deletant mice had ∼50% reduction in baseline and stimulated secretion of mucins (44).

Although it is a crude classification (61, 62), there are many remarkable differences between mucosal and connective tissue MCs (22, 23). Compared with connective tissue MCs, mucosal MCs are smaller, more heterogeneous in shape, less granular, and contain less histamine. Granules of mucosal MCs store chondroitin sulfate proteoglycans and mMCP-1 and -2, while those of connective tissue MCs contain heparin proteoglycans and mMCP-4, -5, -6, and -7 (23, 42, 56).

Something that it is not known is if both MC subpopulations are required and/or sufficient for systemic anaphylaxis. This could not be addressed by using *Kit*^*W-sh/W-sh*^ or *Kit*^*W/W-v*^ mice, which lack both types of MCs (63, 64). The conditional deletion strategy we used removed connective tissue MCs but left intact mucosal MC numbers and their ability to expand upon antigenic stimuli (Fig. 8). When we subjected mice to a systemic anaphylaxis model, we observed that SNAP23^mΔ/Δ^ mice were protected, behaving more like nonsensitized control animals or MC-deficient mice (Fig. 9). This indicates that connective tissue MCs are required and mucosal MCs are insufficient to induce an anaphylactic response.

We have previously reported that regulated exocytosis is barely detectable in MCs lacking Munc13-4 and Munc18-2. Mice with selective MC deletion of those two proteins have normal numbers of mucosal and connective tissue MCs but have a reduced response in the same model of anaphylaxis (6, 7). Nonetheless, there was a residual response in Munc13-4^mΔ/Δ^ and Munc18-2^mΔ/Δ^ mice compared with MC-deficient and unsensitized B6 mice. We postulated two explanations to the partial phenotype. One was that the partial reaction was due to the lack of Cre-induced recombination and therefore intact exocytosis in mucosal MCs. The other was that the residual response was due to a nonexocytic MC effector response. Our current results refute the first hypothesis, because normal numbers of mucosal MCs could not elicit even a partial response in SNAP23^mΔ/Δ^ mice.

Our findings differ from those using administration of diphtheria toxin (DT) to mice expressing the DT receptor in connective tissue MCs, in which there is a reduction but not elimination of peanut-induced anaphylaxis (65). This could be due to the use of a different anaphylaxis model or to a less effective removal of connective tissue MCs by DT. Because using SNAP23^mΔ/Δ^ mice does not require repetitive administrations of DT, it provides a more efficient model to dissect the functions of mucosal MCs from those of connective tissue MCs.

## Experimental procedures

### Mice

We purchased C57BL/6J (B6; catalog n. 000664), MC-deficient B6.Cg-*Kit*^*W-sh*^/HNihrJaeBsmGlliJ (Wsh; n. 012861) (63), and C57BL/6-Tg(Pf4-icre)Q3Rsko/J (n. 008535) (27) from The Jackson Laboratory. We obtained Tg(Cma1-cre)ARoer mice from Dr Axel Roers (Univ. Cologne) (49), and SNAP23 conditional KO mice from Dr Jeffrey E. Pessin (Albert Einstein Coll. Med.) (26). The B6 background of all lines was confirmed using speed congenics.

Exons 3 and 4 of *SNAP23* were flanked by two loxP sites. Cre-mediated recombination induces a frameshift mutation that results in a nonsense mutation and absence of protein expression (26). We crossed SNAP23^F/F^ mice with Tg(Cma1-cre)ARoer to generate MC-specific deletants (SNAP23^mΔ/Δ^) (6, 7, 8), and with C57BL/6-Tg(Pf4-icre)Q3Rsko/J to obtain megakaryocyte/platelet-specific deletants (SNAP23^pΔ/Δ^) (9, 10). Genotyping was done by PCR. Primers to detect the Cma1-cre and Pf4-icre transgenes were the same as before (6, 9). For *SNAP23* we used primers (1) 5′-TCTTTCCAACCCAGAGAGACAC-3′, (2) 5′-GCAACTGCTGGTTTCAAATCT-3′, and (3) 5′-TGCCTCTCATCCCAGTTCAGC-3′, which produced distinct bands for the + allele (981 bp) and F allele (1210 bp). Because tail snips contain dermal MCs and bone marrow megakaryocytes, we could also detect a recombined or − allele (194 bp) in SNAP23^mΔ/Δ^ and SNAP23^pΔ/Δ^ mice.

Breeding of F/F × Δ/Δ mice guaranteed F/F littermates controls for all Δ/Δ mice in all experiments, which included adult animals of both sexes. All studies were carried out using animal protocols approved by the Institutional Animal Care and Use Committee of the University of Texas MD Anderson Cancer Center.

### Sample isolation

For platelet collection, under isoflurane anesthesia, we aspirated ∼800 μl of blood into a citrated syringe (4% Na citrate; 50 μl) by inferior vena cava puncture (21-gauge needle). We processed the samples to obtain whole blood and washed platelets as described (9, 10). Cell counts in these samples were obtained with a VET abc hematology analyzer (Scil) and a Z2 counter (Beckman Coulter).

We collected MCs by peritoneal lavage as described (6, 66). Cells were counted in a Neubauer chamber and cytospins stained with Wright-Giemsa and toluidine blue (pH 0.5). Peritoneal lavage cells were washed with PBS and processed for different assays (see below).

### Primary cell cultures

For PCMCs, we resuspended peritoneal lavage cells in media enriched with rmIL-3 (5 ng/ml) and SCF (15 ng/ml; both from R&D Systems) and cultured them for 2 weeks (37 °C, 5% CO_2_) with biweekly media exchanges.

For BMMCs, bone marrow cells were isolated as described (5, 6) and placed on IL-3 and SCF enriched media as above, but cultured for 6 weeks (37 °C, 5% CO_2_) with biweekly media exchanges.

### Expression studies

For immunoblots, we homogenized and sonicated tissues and cells in cell lysis buffer (6) with protease inhibitors (Sigma-Aldrich). Lysates were run under denaturing conditions on 10% SDS-polyacrylamide gels and transferred to nitrocellulose membranes (Bio-Rad). Blots were probed with anti-SNAP23 (1:5000; Abcam: ab3340), anti-SNAP25 (1:50,000; Synaptic Systems: 111002), anti-SNAP29 (1:10,000; Abcam: ab138500), anti-SNAP47 (1:20,000; Synaptic Systems: 111403), anti-β-actin (1:20,000; Abcam: ab119716), and anti-GAPDH (1:30,000; Abcam: ab9483) antibodies.

### Electron microscopy

Resting and activated (thrombin 0.1 U/ml with 0.7 mM CaCl_2_; 3 min) washed platelets were fixed in Na-cacodylate/glutaraldehyde buffer, postfixed in OsO4 solution, pelleted, and embedded first in low-melting agarose and then in Embed 812 resin, all as described (9, 10). We stained 100 nm-sections with uranyl acetate and lead citrate prior to acquiring images with a Tecnai 12 (FEI) transmission electron microscope (8200×, 100 KeV).

### Stereology

We used ∼20 EM sections per sample with a median of 24 platelets, 20 alpha granules, and 9 dense granule profiles per section (not every platelet profile contained alpha or dense granule profiles). Stereology measurements were performed in STEPanizer (67) using an 81-horizontal line pair grid (line width = 2 pixels [31.8 nm], T-bar = 5 pixels [79.5 μm]) to do point counts and line intercept counts to obtain Vv and Sv, respectively (5, 9, 39).

To measure MOV, we used Gundersen’s approach to point-sample intercepts and nucleator principles (29, 68, 69). Platelet and granule EM profiles were sampled in an unbiased manner using a point grid. For every point that overlapped with an object of interest, a line probe was dropped across the object at a random angle relative to a point near the center, and the length of the line overlapping the object was measured. The mean of all the lengths from one section ($\bar{l}$) was used to calculate: $\text{MOV}={\bar{l}}^{3}\text{π}/3$. Finally, MOVs from all sections of the same genotype were averaged.

### Flow cytometry, cell sorting, and fluorimetry

We labeled proteins on the surface of resting and activated platelets in whole-blood or washed platelet samples. Accucheck counting beads (ThermoFisher) were added to whole-blood samples to calculate the initial volume and concentration of cells. For some experiments, washed platelets were fixed and permeabilized (formaldehyde 1% plus methanol 70%, 10 min, on ice). For activation we used thrombin (0.1 U/ml), ADP (10 μM), and/or collagen (10 μg/ml; all from Chronolog) for 10 min in the presence of CaCl_2_ (0.7 mM).

We labeled 40 μl of whole blood or 2.5 × 10^6^ washed platelets in 40 μl of PBS with 10 μg/ml of antibodies: FITC-anti-P-selectin (BD Pharmingen: RB40.34), FITC-anti-LAMP-1 (BD Pharmingen: 1D4B), PE-anti-α_IIb_β_3_ (Emfret Analytics: Jon/A M023-2), APC-anti-CD41 (eBioscience: 17-0411-80), and anti-PAR4 (Invitrogen: PA5-17408) linked to AlexaF488 (Alexa Fluor 488 protein labeling kit; Invitrogen). All samples were placed on ice, diluted with 1 ml of PBS, and analyzed by flow cytometry (LSR II; BD Biosciences), recording the mean fluorescence intensity (MFI) and calculating the difference between baseline and stimulated MFI (ΔMFI).

We loaded 5 × 10^7^ resting washed platelets with Fura-2-acetoxymethyl ester (Fura-2 AM; 4 μM; Invitrogen) in 1 ml of Tyrode’s buffer with 0.7 mM CaCl_2_ for 45 min, and measured [Ca^2+^]_i_ in a fluorimeter by ratiometry (70, 71) at baseline and for 4 min after activation (thrombin 0.1). We recorded the peak [Ca^2+^]_i_ and the area under the curve (AUC).

For MCs, we incubated 10^6^ peritoneal lavage cells or PCMCs with PE/Cy7-anti-CD117 (200 ng; eBioscience: 25-1171-81) and APC-anti-FcεRIα (200 ng; eBioscience: 17-5898-82) in 100 μl of PBS for 25 min, and analyzed them, recording the number of CD117^+^/FcεRIα^+^ double-positive cells. For sorting, we collected the CD117^+^/FcεRIα^+^ double-positive cells (BD FACSAria).

### Platelet ATP secretion assay

We assessed platelet ATP release by stirring (1200 rpm, 37 °C, 5 min; 700 Lumi-Aggregometer) whole blood (600 μl diluted 5-fold in Tyrode’s buffer) in the presence of luciferin/luciferase and thrombin (0.1 U/ml), collagen (10 μg/ml), or ionomycin (10 μM).

Since SNAP23^pΔ/Δ^ mice were thrombocytopenic (∼40% of +/+ platelet count), samples from +/+, F/F and pΔ/+ mice were diluted ∼1:2.5 to compensate for this. An undiluted +/+ sample was included as control.

### MC secretion assay

We incubated 3 × 10^4^ PCMCs with 100 ng/ml SPE-7 anti-DNP IgE (Sigma-Aldrich) for 5 h, stimulated them with DNP conjugated to human serum albumin (DNP-HSA) or PMA (50 ng/ml) with ionomycin (1 μM) for 30 min, and then measured β-hexosaminidase activity in supernatants and cell lysates as described (7).

### Histology

We harvested several tissues, including ears and small intestine, fixed them in 4% paraformaldehyde (pH 7.0; overnight, 4 °C), and processed them for histology. Paraffin-embedded 5-μm ear sections were labeled with FITC-avidin and Hoechst 33342 (Thermo Fisher Scientific/Molecular Probes) as described (39). We identified MCs as Hoechst^+^ nuclei surrounded by FITC-avidin^+^ granules, and the dermis as the area between the epidermis and the auricular cartilage, and then reported the number of MCs per area of dermis.

Sections of small intestine (5 μm) were stained using Naphthol AS-D Chloroacetate Specific Esterase Kit (Sigma-Aldrich). MCs were identified by size, mononuclear shape, and intense reactivity of CAE compared with polymorphonuclear cells (62, 72). We counted MCs luminal and conterluminal to the basement membrane (bm) as mucosal/intraepithelial and submucosal/connective tissue MCs, respectively. MCs overlapping the bm were counted as connective tissue MCs. We then normalized their numbers to the area of bm (= length of bm × thickness of the section). Using a point grid, we also obtained the Vv of MCs per volume of epithelium or connective tissue using again the bm as the boundary.

### MC electrophysiology

We obtained single MC capacitance recordings employing the same buffers and solutions described before (7, 8). Whole-cell recordings from individual MCs were made using 5 to 6 MΩ patch pipettes coated with dental wax. The internal solution defined [Ca^2+^]_i_, which was monitored ratiometrically with Fura-2 (8), and induced degranulation. For recordings of *C*_m_, membrane conductance (*G*_m_) and series conductance (*G*_s_), an 800 Hz sinusoidal, 30 mV peak-to-peak stimulus was applied around a holding potential of −70 mV, and the resultant signal analyzed using the Lindau–Neher technique (47). For each 100 ms sweep, the average value was recorded, yielding a temporal resolution for *C*_m_, *G*_m_, and *G*_s_ of ∼7 Hz. Cells selected for analysis met the criteria of *G*_m_ ≤ 1200 pS, *G*_s_ ≥ 35 nS and steady-state [Ca^2+^]_i_ = 400 ± 100 nM. Δ*C*_m_, rates of Δ*C*_m_ from 40% to 60% of total Δ*C*_m_, time interval between cell access and exocytic burst, and size and number of *C*_m_ steps between 1% and 15% of total Δ*C*_m_ were obtained as described (6).

### Food allergy model

We used 15 to 20 week-old mice prefasted for 3 to 4 h. We administered by gavage saline (300 μl) alone, with CTX (20 μg; Sigma-Aldrich: C8052), or with CXT and OVA (1 mg; Sigma-Aldrich: A5253) on days 1, 8, 15, and 22, and euthanized them on day 29 (51). Tissues were collected and processed for histology.

### Passive systemic anaphylaxis

We sensitized 15 to 18 week-old mice with SPE-7 anti-DNP IgE i.p. (10 μg in 200 μl of PBS). Controls received only PBS. Next day, we challenged the mice with DNP-HSA i.p. (500 μg in 200 μl of PBS). We monitored the basal core body temperature over time with a rectal thermometer probe (Sper Scientific) and euthanized the mice after 90 min.

To measure plasma histamine, we sensitized and challenged mice as above. At 15 min, we collected blood samples by inferior vena cava puncture (7) avoiding platelet activation, which can cause spurious rises in histamine levels. We separated the plasma and measured histamine concentrations by ELISA (Cayman Biomedical) (6).

### Statistical analysis

For *n* < 7, data is summarized as means ± S.E. (standard error), otherwise we use mean and 5th, 25th, 75th, and 95th percentiles. For continuous variables, we first tested for normality with D’Agostino’s K^2^ test. For normal data, we first compared the means of all groups by ANOVA and, if a significant difference was found, we applied Tukey’s HSD test for multiple pair-wise comparisons, Dunnett’s test for multiple comparisons against a single control group, or Student’s *t*-test for single comparisons. For nonnormal data, we first compared all groups using Kruskal–Wallis *H* test and followed any significant result with Dunn’s test for multiple comparisons or Mann–Whitney *U* test for single comparisons. For categorical data, we used Pearson’s χ^2^ test or Fisher’s exact test. We analyzed frequency distributions with the Kolmogorov–Smirnov test. Significance was set at *p* < 0.05.

## Data availability

All data are contained within the article and supporting information.

## Conflict of interest

The authors declare that they have no conflicts of interest with the contents of this article.

## References

[1] Galli S.J., Tsai M. (2010). Mast cells in allergy and infection: Versatile effector and regulatory cells in innate and adaptive immunity. Eur. J. Immunol..

[2] Kerris E.W.J., Hoptay C., Calderon T., Freishtat R.J. (2020). Platelets and platelet extracellular vesicles in hemostasis and sepsis. J. Investig. Med..

[3] Chen Y.A., Scheller R.H. (2001). SNARE-mediated membrane fusion. Nat. Rev. Mol. Cell Biol..

[4] Rizo J. (2018). Mechanism of neurotransmitter release coming into focus. Protein Sci..

[5] Melicoff E., Sansores-Garcia L., Gomez A., Moreira D.C., Datta P., Thakur P., Petrova Y., Siddiqi T., Murthy J.N., Dickey B.F., Heidelberger R., Adachi R. (2009). Synaptotagmin-2 controls regulated exocytosis but not other secretory responses of mast cells. J. Biol. Chem..

[6] Rodarte E.M., Ramos M.A., Davalos A.J., Moreira D.C., Moreno D.S., Cardenas E.I., Rodarte A.I., Petrova Y., Molina S., Rendon L.E., Sanchez E., Breaux K., Tortoriello A., Manllo J., Gonzalez E.A. (2018). Munc13 proteins control regulated exocytosis in mast cells. J. Biol. Chem..

[7] Gutierrez B.A., Chavez M.A., Rodarte A.I., Ramos M.A., Dominguez A., Petrova Y., Davalos A.J., Costa R.M., Elizondo R., Tuvim M.J., Dickey B.F., Burns A.R., Heidelberger R., Adachi R. (2018). Munc18-2, but not Munc18-1 or Munc18-3, controls compound and single-vesicle-regulated exocytosis in mast cells. J. Biol. Chem..

[8] Sanchez E., Gonzalez E.A., Moreno D.S., Cardenas R.A., Ramos M.A., Davalos A.J., Manllo J., Rodarte A.I., Petrova Y., Moreira D.C., Chavez M.A., Tortoriello A., Lara A., Gutierrez B.A., Burns A.R. (2019). Syntaxin 3, but not syntaxin 4, is required for mast cell-regulated exocytosis, where it plays a primary role mediating compound exocytosis. J. Biol. Chem..

[9] Cardenas E.I., Breaux K., Da Q., Flores J.R., Ramos M.A., Tuvim M.J., Burns A.R., Rumbaut R.E., Adachi R. (2018). Platelet Munc13-4 regulates hemostasis, thrombosis and airway inflammation. Haematologica.

[10] Cardenas E.I., Gonzalez R., Breaux K., Da Q., Gutierrez B.A., Ramos M.A., Cardenas R.A., Burns A.R., Rumbaut R.E., Adachi R. (2019). Munc18-2, but not Munc18-1 or Munc18-3, regulates platelet exocytosis, hemostasis, and thrombosis. J. Biol. Chem..

[11] Kadkova A., Radecke J., Sorensen J.B. (2019). The SNAP-25 protein family. Neuroscience.

[12] Greaves J., Gorleku O.A., Salaun C., Chamberlain L.H. (2010). Palmitoylation of the SNAP25 protein family: Specificity and regulation by DHHC palmitoyl transferases. J. Biol. Chem..

[13] Steegmaier M., Yang B., Yoo J.S., Huang B., Shen M., Yu S., Luo Y., Scheller R.H. (1998). Three novel proteins of the syntaxin/SNAP-25 family. J. Biol. Chem..

[14] Ravichandran V., Chawla A., Roche P.A. (1996). Identification of a novel syntaxin- and synaptobrevin/VAMP-binding protein, SNAP-23, expressed in non-neuronal tissues. J. Biol. Chem..

[15] Wong P.P., Daneman N., Volchuk A., Lassam N., Wilson M.C., Klip A., Trimble W.S. (1997). Tissue distribution of SNAP-23 and its subcellular localization in 3T3-L1 cells. Biochem. Biophys. Res. Commun..

[16] Boschert U., O'Shaughnessy C., Dickinson R., Tessari M., Bendotti C., Catsicas S., Pich E.M. (1996). Developmental and plasticity-related differential expression of two SNAP-25 isoforms in the rat brain. J. Comp. Neurol..

[17] Roth D., Burgoyne R.D. (1994). SNAP-25 is present in a SNARE complex in adrenal chromaffin cells. FEBS Lett..

[18] Itakura E., Kishi-Itakura C., Mizushima N. (2012). The hairpin-type tail-anchored SNARE syntaxin 17 targets to autophagosomes for fusion with endosomes/lysosomes. Cell.

[19] Holt M., Varoqueaux F., Wiederhold K., Takamori S., Urlaub H., Fasshauer D., Jahn R. (2006). Identification of SNAP-47, a novel Qbc-SNARE with ubiquitous expression. J. Biol. Chem..

[20] Kuster A., Nola S., Dingli F., Vacca B., Gauchy C., Beaujouan J.C., Nunez M., Moncion T., Loew D., Formstecher E., Galli T., Proux-Gillardeaux V. (2015). The Q-soluble N-ethylmaleimide-sensitive factor attachment protein receptor (Q-SNARE) SNAP-47 regulates trafficking of selected vesicle-associated membrane proteins (VAMPs). J. Biol. Chem..

[21] Williams C.M., Li Y., Brown E., Poole A.W. (2018). Platelet-specific deletion of SNAP23 ablates granule secretion, substantially inhibiting arterial and venous thrombosis in mice. Blood Adv..

[22] Kitamura Y., Oboki K., Ito A. (2007). Development of mast cells. Proc. Jpn. Acad. Ser. B Phys. Biol. Sci..

[23] Xing W., Austen K.F., Gurish M.F., Jones T.G. (2011). Protease phenotype of constitutive connective tissue and of induced mucosal mast cells in mice is regulated by the tissue. Proc. Natl. Acad. Sci. U. S. A..

[24] Krystel-Whittemore M., Dileepan K.N., Wood J.G. (2015). Mast cell: A multi-functional master cell. Front. Immunol..

[25] Irani A.A., Schechter N.M., Craig S.S., DeBlois G., Schwartz L.B. (1986). Two types of human mast cells that have distinct neutral protease compositions. Proc. Natl. Acad. Sci. U. S. A..

[26] Feng D., Amgalan D., Singh R., Wei J., Wen J., Wei T.P., McGraw T.E., Kitsis R.N., Pessin J.E. (2018). SNAP23 regulates BAX-dependent adipocyte programmed cell death independently of canonical macroautophagy. J. Clin. Invest..

[27] Tiedt R., Schomber T., Hao-Shen H., Skoda R.C. (2007). Pf4-Cre transgenic mice allow the generation of lineage-restricted gene knockouts for studying megakaryocyte and platelet function *in vivo*. Blood.

[28] Kim K., Petrova Y.M., Scott B.L., Nigam R., Agrawal A., Evans C.M., Azzegagh Z., Gomez A., Rodarte E.M., Olkkonen V.M., Bagirzadeh R., Piccotti L., Ren B., Yoon J.H., McNew J.A. (2012). Munc18b is an essential gene in mice whose expression is limiting for secretion by airway epithelial and mast cells. Biochem. J..

[29] Mouton P.R. (2011). Unbiased Stereology: A Concise Guide.

[30] Schneider J.P., Ochs M. (2013). Stereology of the lung. Methods Cell Biol..

[31] French S.L., Hamilton J.R. (2018). Perinatal lethality of Par4(-/-) mice delivered by primiparous dams reveals spontaneous bleeding in mice without platelet thrombin receptor function. Platelets.

[32] Phillips D.R., Charo I.F., Scarborough R.M. (1991). GPIIb-IIIa: The responsive integrin. Cell.

[33] Bergmeier W., Schulte V., Brockhoff G., Bier U., Zirngibl H., Nieswandt B. (2002). Flow cytometric detection of activated mouse integrin alphaIIbbeta3 with a novel monoclonal antibody. Cytometry.

[34] McNicol A., Israels S.J. (1999). Platelet dense granules: Structure, function and implications for haemostasis. Thromb. Res..

[35] Blair P., Flaumenhaft R. (2009). Platelet alpha-granules: Basic biology and clinical correlates. Blood Rev..

[36] Fukuda M. (1991). Lysosomal membrane glycoproteins. Structure, biosynthesis, and intracellular trafficking. J. Biol. Chem..

[37] Warren B.A. (1971). The platelet pseudopodium and its involvement in aggregation and adhesion to vessel walls. Br. J. Exp. Pathol..

[38] Stevens R.L., Adachi R. (2007). Protease-proteoglycan complexes of mouse and human mast cells and importance of their beta-tryptase-heparin complexes in inflammation and innate immunity. Immunol. Rev..

[39] Thakurdas S.M., Melicoff E., Sansores-Garcia L., Moreira D.C., Petrova Y., Stevens R.L., Adachi R. (2007). The mast cell-restricted tryptase mMCP-6 has a critical immunoprotective role in bacterial infections. J. Biol. Chem..

[40] Arinobu Y., Iwasaki H., Gurish M.F., Mizuno S., Shigematsu H., Ozawa H., Tenen D.G., Austen K.F., Akashi K. (2005). Developmental checkpoints of the basophil/mast cell lineages in adult murine hematopoiesis. Proc. Natl. Acad. Sci. U. S. A..

[41] Malbec O., Roget K., Schiffer C., Iannascoli B., Dumas A.R., Arock M., Daeron M. (2007). Peritoneal cell-derived mast cells: An *in vitro* model of mature serosal-type mouse mast cells. J. Immunol..

[42] Nakano T., Sonoda T., Hayashi C., Yamatodani A., Kanayama Y., Yamamura T., Asai H., Yonezawa T., Kitamura Y., Galli S.J. (1985). Fate of bone marrow-derived cultured mast cells after intracutaneous, intraperitoneal, and intravenous transfer into genetically mast cell-deficient W/Wv mice. Evidence that cultured mast cells can give rise to both connective tissue type and mucosal mast cells. J. Exp. Med..

[43] Otsu K., Nakano T., Kanakura Y., Asai H., Katz H.R., Austen K.F., Stevens R.L., Galli S.J., Kitamura Y. (1987). Phenotypic changes of bone marrow-derived mast cells after intraperitoneal transfer into W/Wv mice that are genetically deficient in mast cells. J. Exp. Med..

[44] Ren B., Azzegagh Z., Jaramillo A.M., Zhu Y., Pardo-Saganta A., Bagirzadeh R., Flores J.R., Han W., Tang Y.J., Tu J., Alanis D.M., Evans C.M., Guindani M., Roche P.A., Rajagopal J. (2015). SNAP23 is selectively expressed in airway secretory cells and mediates baseline and stimulated mucin secretion. Biosci. Rep..

[45] Fernandez J.M., Neher E., Gomperts B.D. (1984). Capacitance measurements reveal stepwise fusion events in degranulating mast cells. Nature.

[46] Alvarez de Toledo G., Fernandez J.M. (1990). Patch-clamp measurements reveal multimodal distribution of granule sizes in rat mast cells. J. Cell Biol..

[47] Lindau M., Neher E. (1988). Patch-clamp techniques for time-resolved capacitance measurements in single cells. Pflugers Arch..

[48] Alvarez de Toledo G., Fernandez J.M. (1990). Compound versus multigranular exocytosis in peritoneal mast cells. J. Gen. Physiol..

[49] Scholten J., Hartmann K., Gerbaulet A., Krieg T., Muller W., Testa G., Roers A. (2008). Mast cell-specific Cre/loxP-mediated recombination *in vivo*. Transgenic Res..

[50] Peschke K., Dudeck A., Rabenhorst A., Hartmann K., Roers A. (2015). Cre/loxP-based mouse models of mast cell deficiency and mast cell-specific gene inactivation. Methods Mol. Biol..

[51] Liu Z.Q., Yang G., Geng X.R., Liu J.Q., Mo L.H., Liu Z.G., Yang P.C. (2016). Micro RNA-17-92 cluster mediates interleukin-4-suppressed IL-10 expression in B cells. Am. J. Transl. Res..

[52] Dombrowicz D., Flamand V., Miyajima I., Ravetch J.V., Galli S.J., Kinet J.P. (1997). Absence of Fc epsilonRI alpha chain results in upregulation of Fc gammaRIII-dependent mast cell degranulation and anaphylaxis. Evidence of competition between Fc epsilonRI and Fc gammaRIII for limiting amounts of FcR beta and gamma chains. J. Clin. Invest..

[53] Burgess T.L., Kelly R.B. (1987). Constitutive and regulated secretion of proteins. Annu. Rev. Cell Biol..

[54] Morgan A. (1995). Exocytosis. Essays Biochem..

[55] McNeil H.P., Austen K.F., Somerville L.L., Gurish M.F., Stevens R.L. (1991). Molecular cloning of the mouse mast cell protease-5 gene. A novel secretory granule protease expressed early in the differentiation of serosal mast cells. J. Biol. Chem..

[56] Gurish M.F., Ghildyal N., McNeil H.P., Austen K.F., Gillis S., Stevens R.L. (1992). Differential expression of secretory granule proteases in mouse mast cells exposed to interleukin 3 and c-kit ligand. J. Exp. Med..

[57] Mehlmann L.M., Uliasz T.F., Lowther K.M. (2019). SNAP23 is required for constitutive and regulated exocytosis in mouse oocytesdagger. Biol. Reprod..

[58] Hepp R., Puri N., Hohenstein A.C., Crawford G.L., Whiteheart S.W., Roche P.A. (2005). Phosphorylation of SNAP-23 regulates exocytosis from mast cells. J. Biol. Chem..

[59] Naskar P., Puri N. (2017). Phosphorylation of SNAP-23 regulates its dynamic membrane association during mast cell exocytosis. Biol. Open.

[60] Agarwal V., Naskar P., Agasti S., Khurana G.K., Vishwakarma P., Lynn A.M., Roche P.A., Puri N. (2019). The cysteine-rich domain of synaptosomal-associated protein of 23kDa (SNAP-23) regulates its membrane association and regulated exocytosis from mast cells. Biochim. Biophys. Acta Mol. Cell Res..

[61] Reynolds D.S., Stevens R.L., Lane W.S., Carr M.H., Austen K.F., Serafin W.E. (1990). Different mouse mast cell populations express various combinations of at least six distinct mast cell serine proteases. Proc. Natl. Acad. Sci. U. S. A..

[62] Friend D.S., Ghildyal N., Austen K.F., Gurish M.F., Matsumoto R., Stevens R.L. (1996). Mast cells that reside at different locations in the jejunum of mice infected with Trichinella spiralis exhibit sequential changes in their granule ultrastructure and chymase phenotype. J. Cell Biol..

[63] Grimbaldeston M.A., Chen C.C., Piliponsky A.M., Tsai M., Tam S.Y., Galli S.J. (2005). Mast cell-deficient W-sash c-kit mutant Kit W-sh/W-sh mice as a model for investigating mast cell biology *in vivo*. Am. J. Pathol..

[64] Kitamura Y., Go S., Hatanaka K. (1978). Decrease of mast cells in W/Wv mice and their increase by bone marrow transplantation. Blood.

[65] Reber L.L., Marichal T., Mukai K., Kita Y., Tokuoka S.M., Roers A., Hartmann K., Karasuyama H., Nadeau K.C., Tsai M., Galli S.J. (2013). Selective ablation of mast cells or basophils reduces peanut-induced anaphylaxis in mice. J. Allergy Clin. Immunol..

[66] Adachi R., Krilis S.A., Nigrovic P.A., Hamilton M.J., Chung K., Thakurdas S.M., Boyce J.A., Anderson P., Stevens R.L. (2012). Ras guanine nucleotide-releasing protein-4 (RasGRP4) involvement in experimental arthritis and colitis. J. Biol. Chem..

[67] Tschanz S.A., Burri P.H., Weibel E.R. (2011). A simple tool for stereological assessment of digital images: The STEPanizer. J. Microsc..

[68] Sorensen F.B. (1989). Stereological estimation of nuclear volume in benign melanocytic lesions and cutaneous malignant melanomas. Am. J. Dermatopathol..

[69] Gundersen H.J. (1988). The nucleator. J. Microsc..

[70] Schaeffer J., Blaustein M.P. (1989). Platelet free calcium concentrations measured with fura-2 are influenced by the transmembrane sodium gradient. Cell Calcium.

[71] Sage S.O., Rink T.J. (1987). The kinetics of changes in intracellular calcium concentration in fura-2-loaded human platelets. J. Biol. Chem..

[72] Hamilton M.J., Sinnamon M.J., Lyng G.D., Glickman J.N., Wang X., Xing W., Krilis S.A., Blumberg R.S., Adachi R., Lee D.M., Stevens R.L. (2011). Essential role for mast cell tryptase in acute experimental colitis. Proc. Natl. Acad. Sci. U. S. A..

