# Supplementary material for: SNAP23 is essential for platelet and mast cell development and required in connective tissue mast cells for anaphylaxis
Source: J Biol Chem. 2021 Jan 8;296:100268. doi: 10.1016/j.jbc.2021.100268 (PMC7948755; doi:10.1016/j.jbc.2021.100268)
Supplement: Supplemental Figures S1 and S2 [file mmc1.pdf]

SNAP23 is essential for platelet and mast cell development, and required in connective tissue mast cells for anaphylaxis

Rodolfo A. Cardenas[1,2], Ricardo Gonzalez[3], Elizabeth Sanchez[1], Marco A. Ramos[1], Eduardo I. Cardenas[1,3], Alejandro I. Rodarte[2], Roberto J. Alcazar-Felix[2], Alejandro Isaza[2], Alan R. Burns[4], Ruth Heidelberger[5], Roberto Adachi[1]

From the [1]Department of Pulmonary Medicine, The University of Texas MD Anderson Cancer Center, Houston, Texas 77030, [2]Tecnologico de Monterrey, Escuela de Medicina y Ciencias de la Salud, Monterrey, Nuevo León 64710, Mexico, [3]Tecnologico de Monterrey, Escuela de Ingenieria y Ciencias, Monterrey, Nuevo Leon 64849, Mexico, [4]College of Optometry, University of Houston, Houston, Texas 77204, and [5]Department of Neurobiology and Anatomy, McGovern Medical School at The University of Texas Health Science Center at Houston, Houston, Texas 77030

SUPPORTING INFORMATION

Supplemental Figures

Figure S1. Complete blood counts from SNAP23 mutant mice...…………………………..………S-2

Figure S2. Administration of cholera toxin alone does not affect the number of intestinal MCs…..........S-3

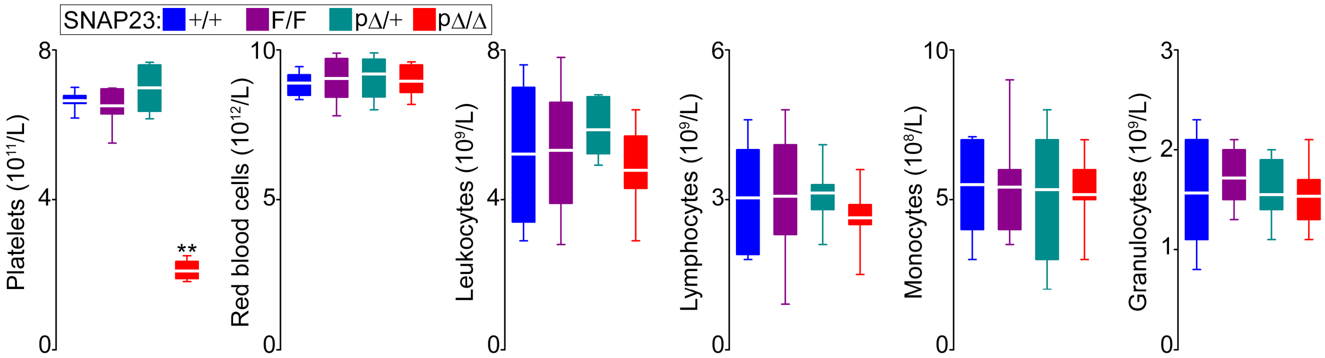

Figure S1. Complete blood counts from SNAP23 mutant mice. Color legend on top applies to all graphs. Platelet, red blood cell, white blood cell and differential counts from whole blood samples obtained with an automated counter. $n = 7$. *White line*, mean; *box*, 25th-75th percentile; *whiskers*, 5th-95th percentile. ** = $p \leq 0.01$; all compared to F/F.

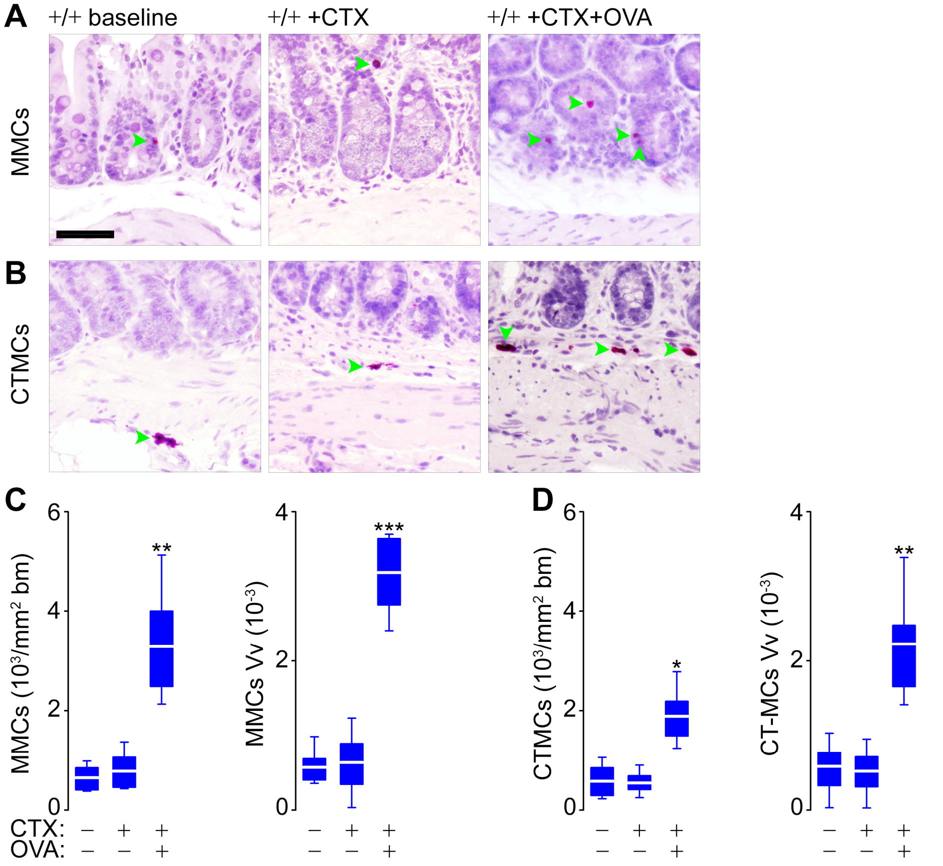

Figure S2. Administration of cholera toxin alone does not affect the number of intestinal MCs. Samples of intestine were collected from B6 mice before and after administration of cholera toxin (CTX) with or without ovalbumin (OVA) by gavage. *A* and *B*, representative histochemical images of mucosal MCs (MMCs) and connective tissue MCs (CTMCs), respectively. *Green arrowheads*, red precipitate indicates CAE activity and labels MCs. *Scale bar* = 50 µm. *C* and *D*, quantification of MCs using the number of MCs per area of basement membrane (bm) and the volume fraction (Vv) of MCs in the host tissue. MCs were identified as mucosal (*C*) or connective tissue (*D*) MCs using the bm as the boundary. n = 21 images from 7 animals. *White line*, mean; *box*, 25th-75th percentile; *whiskers*, 5th-95th percentile. * = $p \leq 0.05$, ** = $p \leq 0.01$, *** = $p \leq 0.001$; all compared to CTX(−) OVA(−).
